# Did the COVID‐19 pandemic increase the long‐term care insurance certification rate in Japan? An interrupted time‐series analysis

**DOI:** 10.1111/ggi.70061

**Published:** 2025-05-14

**Authors:** Satoshi Seino, Toshiki Hata, Hiroki Mori, Shoji Shinkai, Yoshinori Fujiwara, Erika Kobayashi

**Affiliations:** ^1^ Research Team for Social Participation and Healthy Aging, Tokyo Metropolitan Institute for Geriatrics and Gerontology Tokyo Japan; ^2^ Institute of Well‐Being, Yamagata University Yamagata Japan; ^3^ School of Food and Nutritional Sciences, University of Shizuoka Shizuoka Japan; ^4^ Department of Nutrition Sciences Kagawa Nutrition University Saitama Japan; ^5^ Tokyo Metropolitan Institute for Geriatrics and Gerontology Tokyo Japan

Dear Editor,

Previous studies have raised concerns that restrictions on outings and social interactions due to the coronavirus disease 2019 (COVID‐19) pandemic negatively impacted the mental and physical health of older adults, subsequently increasing “pandemic‐associated frailty.”^1^, ^2^ In Japan, the COVID‐19 pandemic did indeed result in a decline in physical activity^3^ and an increase in frailty^1^, ^3^ among older adults. Although these factors might have contributed to an increase in long‐term care insurance (LTCI) certifications,^4^ the comprehensive impact of the pandemic on the nationwide LTCI certification rate remains unclear. Therefore, we analyzed the impact of the COVID‐19 pandemic on the LTCI certification rate in Japan, and present our results here.

We obtained the monthly average LTCI certification rates from the publicly available Monthly Report on the Nationwide Status of LTCI published by the Ministry of Health, Labor and Welfare.^5^ We used data from January 2018 to June 2024 – the most recent period available – because the LTCI certification rate had remained relatively stable before 2018, with a distinct change in trend observed thereafter. Although the state of emergency was officially declared in Japan in April 2020, COVID‐19 had already been significantly impacting the daily lives of the general population well before that time. Therefore, we defined the COVID‐19 pandemic period as beginning in March 2020, after the World Health Organization Director‐General characterized the situation as a pandemic on 11 March 2020.^6^ To compare trends in LTCI certification rates over the 2.2 years before (January 2018 to February 2020) and the 4.3 years (March 2020 to Jun 2024) after the onset of the COVID‐19 pandemic, we applied an interrupted time‐series analysis using segmented regression.^7^ Changes in level and slope of LTCI certifications were estimated, with seasonality adjusted by incorporating Fourier terms with two harmonics (sine and cosine pairs) into the model.^7^, ^8^ Data were analyzed using Stata 18.0 (StataCorp, College Station, TX, USA), and an α of 0.05 indicated statistical significance. The LTCI certification rates used in this study are publicly available and contain no personal information; thus, informed consent was not required, and the study was exempt from ethical review.

Figure 1 presents the results of the interrupted time‐series analysis of the monthly LTCI certification rates. During the study period (January 2018 to June 2024), the LTCI certification rate increased gradually, albeit significantly, from 18.0% (January 2018) to 19.6% (June 2024), at a rate of 0.018 percentage points per month (95% confidence interval [CI] 0.017–0.018). Before the pandemic, the LTCI certification rate showed a significant upward trend of 0.018 percentage points per month (95% CI 0.015–0.020); however, at the onset of the pandemic in March 2020, this trend immediately decreased by a significant amount (−0.092 percentage points per month; 95% CI −0.145 to −0.039). After this sharp decline, the LTCI certification rate showed a slightly stronger upward trend compared with pre‐pandemic levels, at 0.020 percentage points per month (95% CI 0.018–0.021), aligning with the predicted value from January 2024 onward.

**Figure 1:**
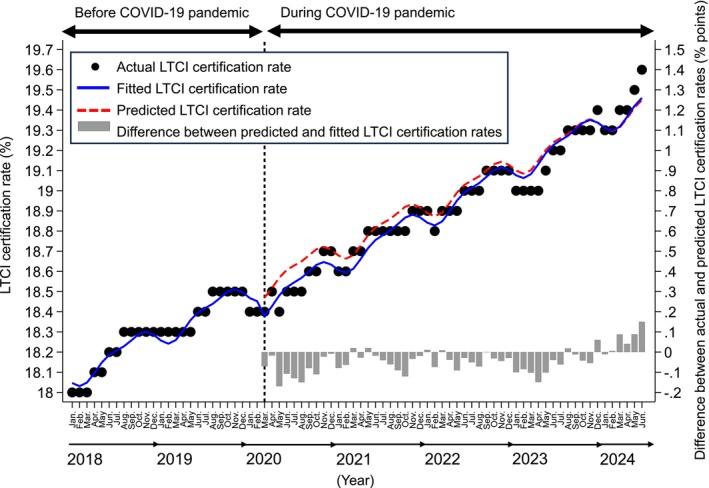
The results of an interrupted time‐series analysis of the monthly long‐term care insurance (LTCI) certification rate in Japan. The solid line represents the regression curve, adjusted for seasonality, for nationwide LTCI certification rates before (January 2018 to February 2020) and during (March 2020 onwards) the coronavirus disease 2019 pandemic. The dotted line indicates predicted values based on the pre‐pandemic regression model.

Contrary to expectations, the COVID‐19 pandemic did not increase the nationwide LTCI certification rate. One possible reason why the certification rate did not rise as much as anticipated, even in this long‐term analysis, is that the early rollout of vaccines might have enabled physical activity and social engagement to partially return to pre‐pandemic levels at an early stage.^2^, ^9^, ^10^

In contrast, the temporary decline in the LTCI certification rate observed immediately after the onset of the pandemic has important implications for public health policy. This decline might have resulted from older adults and their families refraining from initiating the LTCI application process due to concerns about severe acute respiratory syndrome coronavirus 2 (SARS‐CoV‐2) infection.^11^ Indeed, an analysis of the data from a cohort of older adults in Tokyo^12^ showed a trade‐off relationship: during the early stages of the pandemic, the number of LTCI applications decreased as SARS‐CoV‐2 infections increased, and subsequently increased as SARS‐CoV‐2 infections declined. In addition, there have also been reports of cases in which home‐visit assessments and meetings of the Municipal Certification Committee – both essential steps in the LTCI certification process – were disrupted by the pandemic.^11^ As these disruptions might have contributed to the suppression of new LTCI certifications, developing contingency plans to ensure uninterrupted access to LTCI services during public health emergencies remains a critical issue for future policy planning.

In summary, although the national LTCI certification rate immediately experienced a temporary dip at the onset of the pandemic, it subsequently began to trend upward, aligning with the predicted rate based on pre‐pandemic trends from January 2024 onward. Although the impact of the COVID‐19 pandemic on the national LTCI certification rate was statistically significant, the effect size was minimal, showing that the pandemic did not substantially increase the rate. The limitation of this analysis is that we examined the LTCI certification rate based solely on the national average. Regional differences in infection levels and population density might have influenced these results.^10^ Therefore, continued monitoring of national LTCI certification trends and detailed analyses by region and certification type (new vs renewed) are still needed.

## Disclosure statement

The authors declare no conflict of interest.

## Data Availability

Data are available upon reasonable request to the authors.
